# Potential role of conventional and speckle-tracking echocardiography in the screening of structural and functional cardiac abnormalities in elderly individuals: Baseline echocardiographic findings from the LOOP study

**DOI:** 10.1371/journal.pone.0269475

**Published:** 2022-06-03

**Authors:** Flemming Javier Olsen, Søren Zöga Diederichsen, Peter Godsk Jørgensen, Magnus T. Jensen, Anders Dahl, Nino Emmanuel Landler, Claus Graff, Axel Brandes, Derk Krieger, Ketil Haugan, Lars Køber, Søren Højberg, Jesper Hastrup Svendsen, Tor Biering-Sørensen

**Affiliations:** 1 Department of Cardiology, Copenhagen University Hospital—Herlev and Gentofte, Herlev, Denmark; 2 Department of Cardiology, Zealand University Hospital, University of Copenhagen, Roskilde, Denmark; 3 Department of Cardiology, Copenhagen University Hospital—Rigshospitalet, Copenhagen, Denmark; 4 Department of Cardiology, Copenhagen University Hospital—Amager and Hvidovre Hospital, Hvidovre, Denmark; 5 Centre for Advanced Cardiovascular Imaging, William Harvey Research Institute, Queen Mary University of London, London, United Kingdom; 6 Department of Health Science and Technology, Aalborg University, Aalborg, Denmark; 7 Department of Cardiology, Odense University Hospital, Odense, Denmark; 8 Department of Clinical Research, Faculty of Health Sciences, University of Southern Denmark, Odense, Denmark; 9 University Hospital Zurich, University of Zurich, Zürich, Switzerland; 10 Stroke Unit, Mediclinic City Hospital, Dubai, UAE; 11 Department of Clinical Medicine, University of Copenhagen, Copenhagen, Denmark; 12 Department of Cardiology, Copenhagen University Hospital–Frederiksberg and Bispebjerg Hospital, Copenhagen, Denmark; 13 Department of Biomedical Sciences, University of Copenhagen, Copenhagen, Denmark; Charité Universitätsmedizin Berlin - Campus Virchow-Klinikum: Charite Universitatsmedizin Berlin - Campus Virchow-Klinikum, GERMANY

## Abstract

**Background:**

Elderly individuals occupy an increasing part of the general population. Conventional and speckle-tracking transthoracic echocardiography may help guide risk stratification in these individuals. The purpose of this study was to evaluate the potential utility of conventional and speckle-tracking echocardiography in the screening of cardiac abnormalities in the elderly population.

**Methods:**

Two cohorts of elderly individuals (sample size: 1441 and 944) were analyzed, who were part of a randomized controlled clinical trial (LOOP study) and of an observational study (Copenhagen City Heart Study), recruiting participants from the general population >70 years of age with cardiovascular risk factors (arterial hypertension, diabetes mellitus, heart failure, or prior stroke) and sinus rhythm. Participants underwent a comprehensive transthoracic echocardiographic examination, including myocardial speckle tracking. Cardiac abnormalities were defined according to the ASE/EACVI guidelines.

**Results:**

Structural cardiac abnormalities such as left ventricular (LV) remodeling, mitral annular calcification (MAC), and aortic valve sclerosis (with or without stenosis) were highly prevalent in the LOOP study (40%, 39%, and 27%, respectively). Moreover, a high prevalence of functional cardiac alterations such as LV diastolic dysfunction (LVDD), abnormal LV longitudinal systolic strain (GLS), and abnormal left atrial (LA) reservoir strain was present in the LOOP study (27%, 18%, and 9%, respectively). Likewise, the rate of LVDD, abnormal GLS, and abnormal LA reservoir strain was comparable in the validation sample from the Copenhagen City Heart Study. In line with these findings, subjects with LV remodeling, MAC, and aortic valve changes had a higher prevalence of LVDD, abnormal GLS, and abnormal LA reservoir strain than those without structural cardiac alterations.

**Conclusion:**

The findings of this study highlight the potential clinical utility of conventional and speckle-tracking echocardiography in the screening of structural and functional cardiac abnormalities in the elderly population. Further studies are warranted to determine the prognostic relevance of these findings.

## Introduction

Population ageing is a world-wide phenomenon which poses significant challenges to our health care systems and contributes increasingly to cardiovascular disease burden. The World Health Organization estimates that the number of people above 60 years of age will more than double by 2050, and the number of elderly above 80 years of age will more than triple to approximately 450 million worldwide [1]. The ageing population is in part a result of a reduction in cardiovascular mortality through improvements made in preventive, diagnostic and management strategies for cardiovascular disease [2, 3]. However, as age increases, the proportion of age-dependent chronic diseases increases concordantly, and specifically cardiovascular chronic diseases contribute substantially to disability in the elderly [4]. One of the most frequent causes of disability is stroke, and methods to prevent this disease have therefore been of particular interest. As atrial fibrillation (AF) becomes more frequent with age, one focus point has been on improving detection of AF, a frequent and well-known cause of ischemic stroke. The LOOP study recently found that use of continuous rhythm monitoring for subclinical AF with subsequent anticoagulation did not reduce stroke events [5]. Characterizing the cardiac phenotype in the LOOP study may, however, help inform on the type of patients who could benefit from this strategy of screening for AF. It will also provide insight into the association between AF and structural and functional properties of the left atrium (LA), which could deepen our understanding of the electro-mechanical coupling of the LA and the pathophysiology that drives the development of subclinical AF. Studies suggest that echocardiographic measures of LA function may deteriorate prior to volume dilatation and be more closely associated to prevalent and incident clinical AF [6, 7]. A similar link to subclinical AF has also been suggested in retrospective and small prospective studies, however, larger, robust data are lacking [8, 9]. With this in mind, we performed a comprehensive echocardiographic substudy in the LOOP study. Herein, we sought to characterize the echocardiographic phenotype of the participants in the LOOP study, and to describe the extent of structural and functional cardiac abnormalities in such high-risk participants.

## Methods

### Participants

This was an echocardiographic substudy based on an investigator-initiated, multicenter, open-label randomized controlled trial (the LOOP study, Clinicaltrials.gov identifier: NCT02036450). The LOOP study included participants aged 70 years or above with at least one of the following cardiovascular risk factors: hypertension, diabetes mellitus, heart failure, and prior stroke (target population: n = 6,005). Eligible participants were identified from the Danish Nationwide Patient Registry using ICD-10 codes and were recruited by letter invitation to their homes. At the screening visit, experienced research nurses would review the participant’s medical history and medication usage to confirm all comorbidities. Participants who confirmed the presence of the above-mentioned risk factors were eligible for inclusion. Exclusion criteria comprised any history of AF or implanted cardiac electronic devices. Eligible participants were randomized to standard of care (control group) or to receive an implantable loop recorder (ILR) in a 3:1 fashion. In the ILR arm, continuous monitoring was applied in order to diagnose subclinical AF with concomitant oral anticoagulation if episodes lasting ≥6 min were detected. Specific details on the trial design have previously been described [10]. Participants were enrolled at 4 centers in Denmark. Due to geography only participants enrolled from three of the centers were invited for participation in the echocardiographic substudy between May 2014 and November 2017.

Informed consent was obtained from all participants and the study adhered to the 2^nd^ Helsinki Declaration. The study was approved by a regional scientific ethics committee (no.: H-4-2013-025) and the Danish Data Protection Agency (no.: 2007-58-0015).

#### Baseline visit

Upon inclusion, participants were invited for an outpatient baseline visit at the recruiting center. Information on medical history, cardiovascular symptoms, demographics, and assessment of CHA_2_DS_2_-VASc score was acquired. Height, weight, blood pressure, and heart rate were measured.

### Echocardiographic acquisition and analysis

The participants underwent the transthoracic echocardiogram at the Cardiovascular Non-Invasive Imaging Research Laboratory at Gentofte Hospital, Copenhagen, Denmark using a GE Healthcare Vivid E9 ultrasound machine. Initially, only participants randomized to ILR had echocardiography performed, and later the participants in the control group were also invited to have echocardiography performed. This explains why more participants in the ILR group underwent echocardiography as compared to the control group, even though the LOOP study randomized more participants to the control group.

The exams were stored in a remote digital image archive and analyzed offline with post-processing software (EchoPAC BT2.02, GE Healthcare). All echocardiographic analyses were performed by a single analyzer experienced in echocardiographic analyses (FJO). Loops of three cardiac cycles were stored for all images when feasible, and the cardiac cycle with best image quality was chosen for analyses. All echocardiographic analyses were performed according to the most recent EACVI/ASE cardiac chamber quantification recommendations from 2015, and cutoffs for abnormalities were also defined in accordance with these recommendations [11].

#### Left ventricular structure

Left ventricular (LV) dimensions were measured at end-diastole in the parasternal long-axis view and included: interventricular septal wall thickness, LV internal dimension (LVIDd), and posterior wall thickness. These were used to calculate the relative wall thickness (RWT), and the LV mass by Devereux’ formula [12]. LV mass was indexed to body surface area (calculated by DuBois’ formula) to provide the LV mass index (LVMI). Left ventricular hypertrophy (LVH) was defined as an LVMI>95g/m^2^ for women and LVMI>115g/m^2^ for men and subdivided into concentric hypertrophy if RWT>0.42 and eccentric hypertrophy if RWT<0.42. Concentric remodeling was defined as an RWT>0.42 in the absence of LVH. LV dilatation was defined as an indexed LVIDd>3.0 for men and indexed LVIDd>3.1 for women [11].

#### Left ventricular systolic function

Left ventricular ejection fraction (LVEF) was assessed by the Simpson’s biplane method. Mild systolic dysfunction was defined as an LVEF of 40–52% for men and 40–54% for women, moderate systolic dysfunction was defined as LVEF of 30–40%, and severe systolic dysfunction as LVEF<30% [11]. Global longitudinal strain (GLS) was assessed according to proposed recommendations for LV speckle tracking analyses [13]. Speckle tracking was performed in all three apical views. Only images with adequate frame rate (>40 frames/second) were analyzed. Tracing was performed with a semi-automatic approach by placing two samples at the base of the LV walls and one at the apex in each projection. This generated a region of interest encompassing the endocardial layer and throughout the myo-epicardial border. The width of the region of interest could be adjusted at the discretion of the analyzer. Individual segments could be excluded in the presence of artefacts or segment dropout. However, only 1 segment could be excluded per projection, otherwise the image quality was deemed too poor and speckle tracking was considered infeasible. Abnormal myocardial deformation was defined as GLS > -16% [14].

#### Right ventricular function and pressure

Right ventricular systolic function was assessed by the tricuspid annular systolic plane excursion (TAPSE) measured by m-mode from a modified 4-chamber view focused on the right ventricle. Impaired right ventricular systolic function was defined as TAPSE<17mm. A continuous wave Doppler cursor was placed through a tricuspid regurgitant jet if tricuspid regurgitation was present to measure the peak tricuspid regurgitant velocity (TR_Vmax_) as an estimate of right ventricular pressure. Elevated right ventricular pressure was defined as a TR_Vmax_>2.8m/s. Likelihood of pulmonary hypertension was estimated as high probability if TR_Vmax_>3.4m/s and intermediate probability if TR_Vmax_ was between 2.8m/s and 3.4m/s in the absence of pulmonary vascular disease as suggested by guidelines [15].

#### Left atrial size and function

Left atrial (LA) volumes were measured by the Simpson’s biplane method. Volume tracings were performed at end-systole (LAV_max_), at end-diastole (LAV_min_), and at the ECG p-wave (LAV_p-wave_). These were used to calculate LA emptying fractions (LAEF) according to previous reports [16]: ${LAEF}_{total}=\frac{{LAV}_{max}-{LAV}_{min}}{{LAV}_{max}}$

$${LAEF}_{passive}=\frac{{LAV}_{max}–{LAV}_{p-wave}}{{LAV}_{max}}$$

$${LAEF}_{active}=\frac{{LAV}_{p-wave}–{LAV}_{min}}{{LAV}_{p-wave}}$$

Abnormal LA size was defined as LAV_max_>34mL/m^2^. Mildly abnormal LA size was defined as LAV_max_: 34-41mL/m^2^, moderately abnormal as LAV_max_: 42-48mL/m^2^, and severely abnormal as LAV_max_ >48mL/m^2^. Left atrial speckle tracking was performed according to recent recommendations [17]. Again, only images with adequate frame rate were analyzed. Analyses were performed as biplane analyses from the apical 4-chamber and 2-chamber views by R-wave triggering and using the software otherwise dedicated to the LV. Manual delineation of the endocardium was performed after which the system generated a region of interest that could be adjusted at the discretion of the analyzer. If more than 1 segment was untraceable then the entire projection was excluded. The measurements were included from only one projection if the speckle tracking analyses could not be performed in both projections. LA speckle tracking provided the LA reservoir strain (ε_s_), LA conduit strain (ε_e_), and LA contraction strain (ε_a_). A reservoir strain <23% was considered abnormal [18].

#### Diastolic function

Transmitral inflow was assessed by pulsed-wave Doppler imaging with a sample placed at the tips of mitral valve leaflets in the apical 4-chamber view. This was used to measure early transmitral inflow velocity (E-wave), late transmitral inflow velocity (A-wave), E/A-ratio, and E-wave deceleration time. Pulsed-wave tissue Doppler imaging was used to measure the e’ as an average of two samples placed at the base of the inferoseptal and lateral walls. This was indexed to the transmitral E-wave to provide the E/e’. An average E/e’ > 14 was considered abnormal. If only septal e’ was available, then septal E/e’>15 was considered abnormal, and similarly a lateral E/e’>13 was considered abnormal if only lateral e’ was available.

Diastolic dysfunction (DDF) was determined according to the 2016 EACVI/ASE recommendations [19]. Participants were considered to have DDF if they had reduced LVEF (<50%) or myocardial disease. Myocardial disease was defined as: dilated LV, LVH, or abnormal GLS. The other participants were classified as having DDF if they had >50% of the following positive indicators: E/e’ > 14, septal e’ <7cm/s or lateral e’ <10cm/s, TR_Vmax_>2.8m/s, or LAV_max_>34mL/m^2^. If participants had 50% positive indicators then diastolic function was considered indeterminate, whereas normal diastolic function was defined as presence of <50% indicators.

Participants with reduced LVEF, myocardial disease, or DDF by the above-described approach underwent grading of DDF. Grade 1 DDF was defined as 1) E/A≤0.8 and E-wave≤50cm/s or 2) either an E/A≤0.8 + E-wave>50cm/s or an E/A: 0.8–2.0 with none or only a single of the following indicators of elevated filling pressure: E/e’>14, TR_Vmax_>2.8m/s, LAV_max_>34mL/m^2^. Grade 2 DDF was defined as either an E/A≤ 0.8 + E-wave>50cm/s or an E/A: 0.8–2.0 with two or more positive indicators of elevated filling pressure (as listed above). Grading was considered indeterminate if only two indicator variables were available with one being positive and one being negative. Grade 3 DDF was defined as an E/A≥2.0.

#### Valvular assessment

Valvular abnormalities are reported in accordance with guidelines [20, 21]. Mitral regurgitation (MR) was quantified by either vena contracta as assessed from the parasternal long-axis view or by the proximal isovelocity surface area (PISA) method. Cases of trace MR are not reported. When quantification was not technically feasible, particularly when the severity was borderline between trace and mild, a qualitative assessment by the analyzer was made to distinguish trace from mild MR. Mitral stenosis was considered present if the transmitral mean gradient was above 5mmHg by continuous wave Doppler tracing. Aortic regurgitation (AR) was assessed by the vena contracta in the parasternal long-axis view or by the pressure half-time (PHT) from continuous-wave flow profiles in the apical projections. Aortic stenosis (AS) was considered present when the peak velocity by continuous wave Doppler was above 2.5m/s. AS was graded as follows: mild AS: peak velocity <2.9m/s, mean gradient <30mmHg, aortic valve area (AVA)>1.5cm^2^, moderate AS: peak velocity 3-4m/s, mean gradient 30-50mmhg, AVA 1.0–1.5cm^2^, and severe AS: peak velocity>4.0cm/s, mean gradient>50mmHg, and AVA<1.0cm^2^.

### Statistics

Clinical characteristics were compared between participants who had an echocardiogram performed vs. those who did not have an echocardiogram performed, and echocardiographic characteristics were compared between the two randomization groups. A comparison was also made to elderly participants (>70 years of age) with either hypertension, diabetes mellitus, prior stroke, or heart failure and with no history of AF in the 5^th^ Copenhagen City Heart Study (CCHS) (n = 944). Finally, abnormal echocardiographic findings were also compared as stratified by presence of mitral annular calcification. LV functional abnormalities are outlined in participants without coronary artery disease (CAD), in participants with normal LVEF without CAD, and in participants with normal LVEF, normal LA size and no CAD.

Continuous variables showing Gaussian distribution are displayed as mean ± SD, whereas variables not showing Gaussian distribution are shown as median with interquartile ranges [25–75 percentiles]. Categorical variables are displayed in numbers and percentages. For these comparisons, continuous Gaussian-distributed variables were compared using Student’s T-test and non-Gaussian-distributed variables were compared with the Rank-sum test. Participants were also stratified according to categories of CHA_2_DS_2_-VASc score (2–3, 4–5, ≥6). The ANOVA test was used to test for differences across categories for Gaussian-distributed continuous variables, and Kruskal-Wallis was used for non-Gaussian distributed variables. In all analyses, categorical variables were compared between groups by Chi^2^-test or Fisher’s exact test if expected number of observations were below 5 within a group. A Venn diagram was created to illustrate the overlap between abnormal LV geometry and systolic function as well as the overlap between DDF and LA size and function.

A p-value<0.05 was considered significant. All statistical analyses were performed using STATA version 15 (StataCorp LP, College Station, TX).

## Results

**Fig 1:**
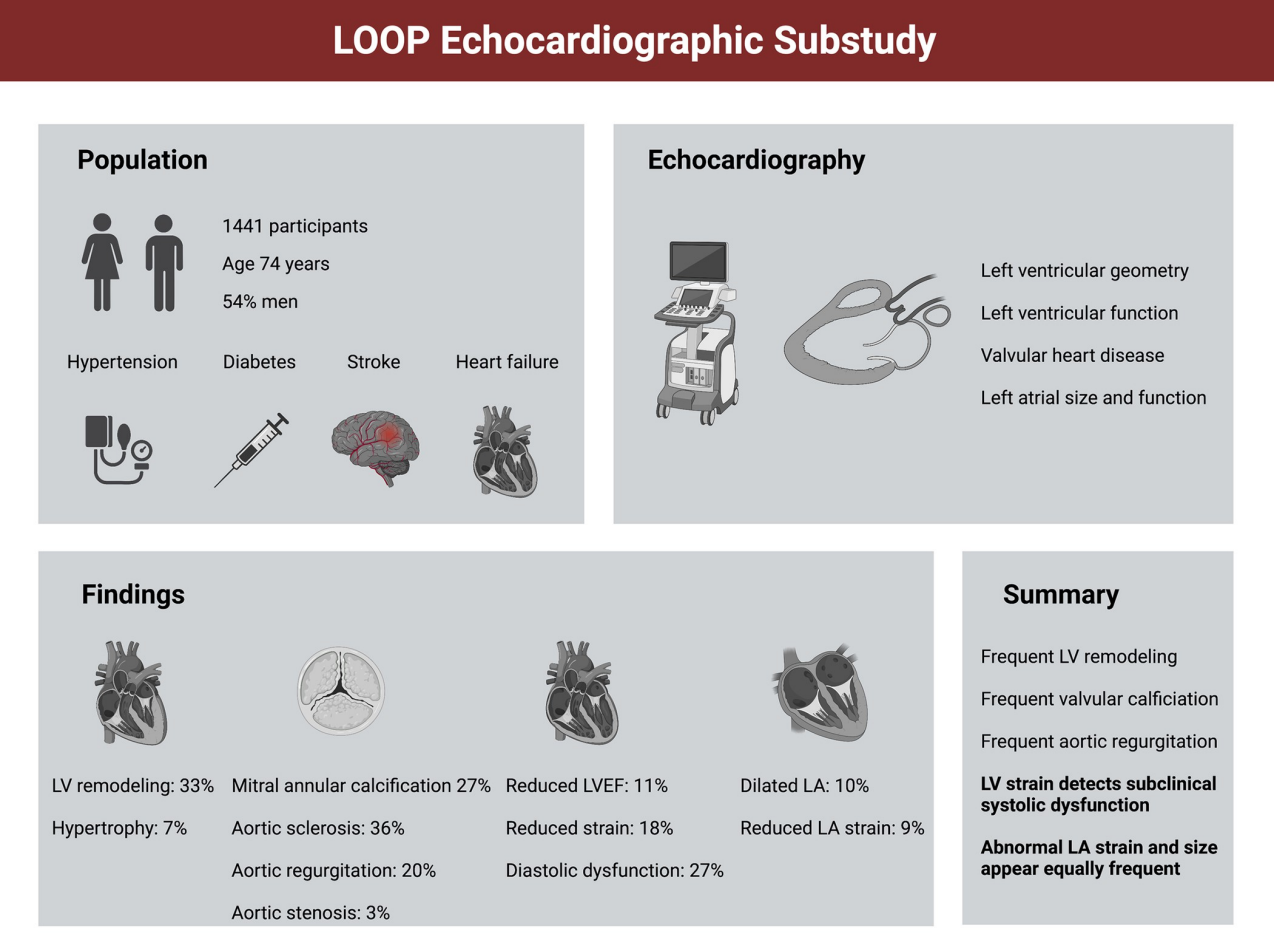
Graphical abstract. A graphical depiction of the study concept and main findings. Created with BioRender.com.

We included 1,441 participants, of whom 1,020 were randomized to have an ILR inserted and 421 were randomized to the control group. Clinical characteristics for the participants in this substudy are shown in Table 1. Briefly, the clinical characteristics of the participants in the echocardiographic substudy were as follows: 783 (54%) were men, mean age of 74±4 years, mean heart rate was 71±12 beats/minute, and mean blood pressure was 150/84mmHg (±19/11). Of cardiovascular risk factors, 1,306 (91%) had hypertension, 421 (29%) had diabetes mellitus, 65 (5%) had heart failure, and 302 (21%) had a prior ischemic stroke and/or systemic embolism. The median CHA_2_DS_2_-VASc score was 4 (IQR: 3–4). A brief summary of the main echocardiographic findings is shown in Fig 1.

**Table 1:** Baseline clinical characteristics of the LOOP study.

	All n = 1,441
Age, years	74.4±4.1
Men, n (%)	784 (54.4)
Smoking pack years	7 [0;28]
Alcohol consumption, units/week	5 [1;11]
Systolic blood pressure, mmHg	149.3±18.9
Diastolic blood pressure, mmHg	84.8±11.1
Height, cm	171.0±8.9
Weight, kg	81.0±15.6
Body mass index, kg/m^2^	27.6±4.5
Heart rate, beats/minute	71.7±12.3
Hypertension, n (%)	1,306 (90.6)
Diabetes mellitus, n (%)	421 (29.2)
Heart failure, n (%)	65 (4.5)
Previous stroke or systemic embolism n (%)	302 (21.0)
Previous myocardial infarction, n (%)	148 (10.3)
Previous CABG, n (%)	86 (6.0)
Known valve disease, n (%)	59 (4.1)
PAD, n (%)	91 (6.3)
COPD, n (%)	88 (6.1)

Gaussian-distributed continuous variables are presented as mean±SD. Non-gaussian-distributed variables are presented as median with interquartile range.

Abbreviations: CABG: coronary artery bypass grafting; PAD: peripheral artery disease; COPD: chronic obstructive pulmonary disease.

A comparison to elderly at-risk participants from the CCHS is shown in Table 2. In terms of clinical features, the participants in the CCHS were older and more frequently had hypertension, but less frequently had diabetes and prior stroke.

**Table 2:** Comparison between LOOP study and Copenhagen City Heart Study.

	LOOP study n = 1,441	5^th^ Copenhagen City Heart Study n = 944
**Clinical**		
Age, years	74±4	77±5
Hypertension, n (%)	1,305 (91)	926 (98)
Diabetes mellitus, n (%)	421 (29)	103 (11)
Prior stroke, n (%)	240 (17)	71 (8)
Heart failure, n (%)	65 (5)	37 (4)
**Echocardiography** *		
Global longitudinal strain, %	-18.3±2.7	-18.5±2.9
Left atrial volume index, mL/m^2^	24 [20;29]	25 [20;31]
Left atrial reservoir strain, %	34±8	32±11
Septal e’, cm/s	6.2±1.6	6.2±1.8
Lateral e’, cm/s	7.9±2.2	8.4±2.6
E/e’	9.2 [7.8;11.2]	9.5 [8.1;12.0]
E/A	0.83 [0.69;1.00]	0.81 [0.70;0.97]
Tricuspid regurgitant velocity, m/s*	2.4±0.3	2.5±0.3
**Abnormalities**		
Global longitudinal strain < 16%	246 (18)	131 (16)
Left atrial volume index > 34 mL/m^2^	149 (10)	127 (14)
Left atrial reservoir strain <23%	130 (9)	138 (19)
Septal e’ <7 cm/s	1,014 (71)	610 (69)
Lateral e’ < 10 cm/s	1,210 (84)	660 (75)
E/e’ > 14	129 (9)	98 (11)
E/A > 2	14 (1)	10 (1)
Tricuspid regurgitant velocity > 2.8 m/s	98 (11)	87 (16)

Gaussian-distributed continuous variables are presented as mean±SD. Non-gaussian-distributed variables are presented as median with interquartile range.

*Available in the CCHS group: GLS (n = 818), LAVi (n = 863), LA strain (n = 723), septal e’ (n = 887), lateral e’ (n = 882), E/e’ (n = 864), E/A (n = 871), TR velocity (n = 536).

Clinical characteristics for participants who had an echocardiogram performed compared to those who did not have an echocardiogram performed are shown in S1 Table. Those included in the echocardiographic substudy were comparable to those not included in this substudy, albeit with minor differences in diastolic blood pressure, height, and age. Additionally, fewer with chronic obstructive pulmonary disease (COPD) were included in this substudy.

Cardiac structural and functional abnormalities stratified by randomization groups are shown in S2 Table. Briefly, the abnormalities were balanced between randomization groups with only a slightly higher proportion of aortic regurgitation observed in the control group as compared to the ILR group.

### Left ventricular structure and systolic function

None of the participants exhibited a dilated LV, however, 572 (40%) had altered LV geometry with 92 (6%) having LVH. Of note, even though participants with hypertension frequently had altered LV geometry, this was not more frequent as compared to non-hypertensive participants (40% vs. 34%, p = 0.16). Participants with LV remodeling more frequently had DDF (37% vs. 21%, p<0.001), abnormal GLS (22% vs. 15%, p<0.001), and a trend towards more frequent abnormal LA strain (11% vs. 8%, p = 0.08).

Mean LVEF was 61±7% and 165 (11%) presented with LV systolic dysfunction. The majority of these participants had mild systolic dysfunction (86%) (S3 Table). Participants with a history of acute myocardial infarction more frequently had systolic dysfunction (29% vs. 9%, p<0.001) and similar observations were noted for participants with known heart failure (46% vs. 10%, p<0.001).

**Fig 2:**
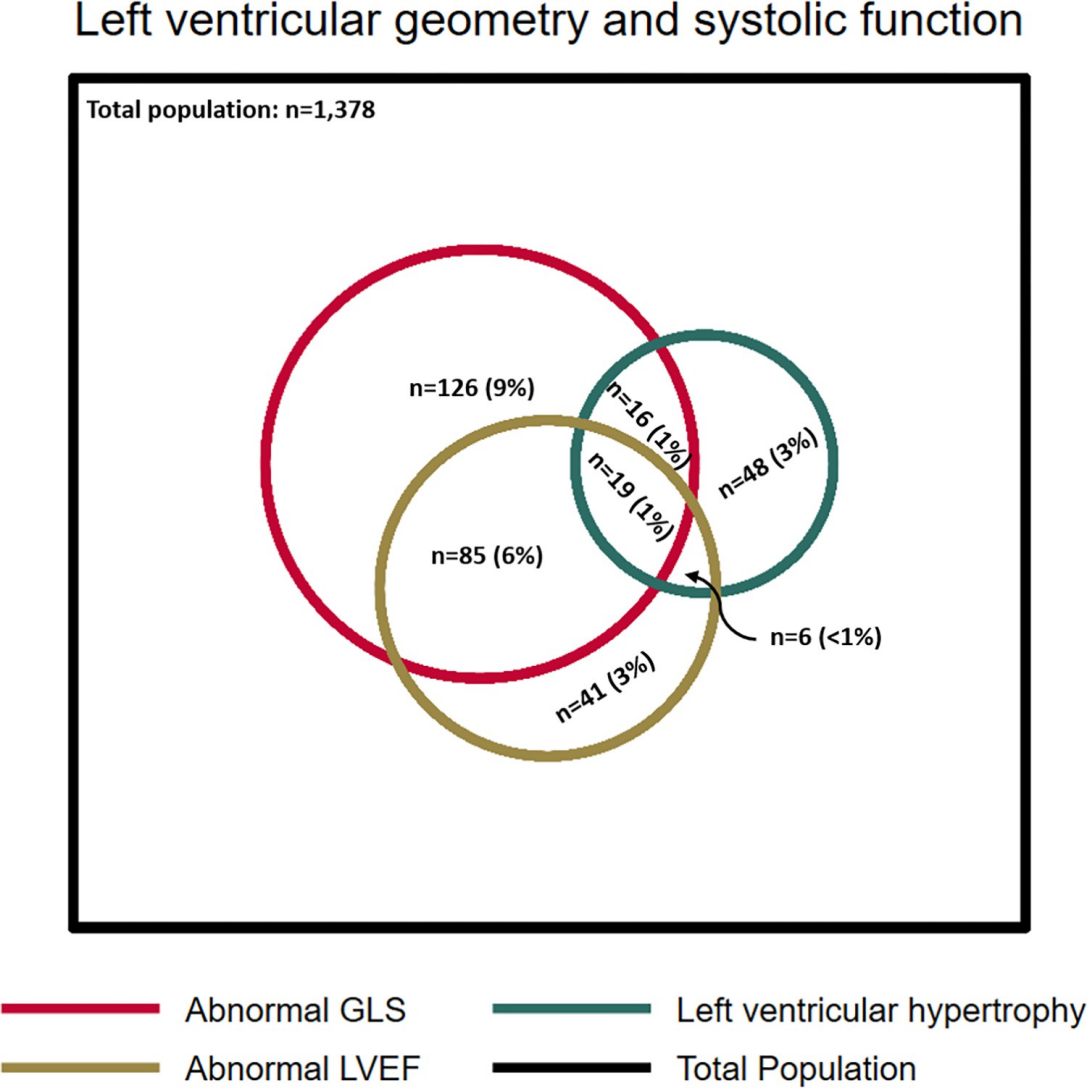
Diagram of abnormal left ventricular geometry and systolic function. The figure shows the prevalence of left ventricular hypertrophy, abnormal GLS, and abnormal LVEF as well as the overlap between the different groups. The percentages represent the proportion according to the total population in whom GLS was obtainable (n = 1,378). GLS: global longitudinal strain, LVEF: left ventricular ejection fraction.

LV speckle tracking analyses was feasible in 1,378 (96%) of the participants. The mean frame rate was 63±5 frames per second. The mean GLS was -18.3±2.7%, comparable to the CCHS (Table 2). Speckle tracking analyses revealed that 246 (18%) had abnormal systolic deformation, slightly more frequent than in the CCHS. Among participants who presented with normal systolic function by LVEF, 142 (12%) had reduced GLS, whereas only 47 (4%) of those with normal GLS exhibited abnormal LVEF. The overlap between abnormalities in LVEF, GLS and LVH is shown in Fig 2.

Even in the subgroup of participants without CAD, abnormal GLS was observed in 14% (Table 3), and in the subgroup who further had preserved LVEF, abnormal GLS was observed in 10% and was balanced across risk groups (Table 4). This was consistent in those who also had normal LA size (Table 5).

**Table 3:** LV functional abnormalities in the population and subgroups without CAD in the LOOP study.

	All without CAD	Hypertension	Diabetes
	n = 1,252	n = 1,131	n = 355
Left ventricular ejection fraction			
• Normal	1,113 (91)	1,024 (91)	307 (87)
• Abnormal	119 (10)	107 (10)	48 (14)
Global longitudinal strain			
• Normal	1,031 (86)	935 (86)	281 (84)
• Abnormal	169 (14)	154 (14)	55 (16)
Septal e’			
• Normal	386 (31)	349 (31)	108 (30)
• Abnormal	865 (69)	781 (69)	247 (70)
Lateral e’			
• Normal	198 (16)	183 (16)	50 (14)
• Abnormal	1,051 (84)	946 (84)	303 (86)
E/e’			
• Normal	1,140 (91)	1,029 (92)	312 (89)
• Abnormal	107 (9)	97 (9)	41 (12)
E/A			
• Normal	1,237 (99)	1,116 (99)	351 (99.7)
• Abnormal	7 (1)	7 (1)	1 (0.3)
Tricuspid regurgitant velocity			
• Normal	721 (89)	650 (89)	168 (88)
• Abnormal	87 (11)	83 (11)	24 (13)
Left atrial volume			
• Normal	1,133 (91)	1,021 (90)	322 (91)
• Abnormal	119 (10)	110 (10)	33 (9)
Left atrial reservoir strain			
• Normal	1,144 (93)	1,031 (92)	321 (92)
• Abnormal	91 (7)	85 (8)	28 (8)

LV: left ventricular; CAD: coronary artery disease.

**Table 4:** LV functional abnormalities in risk groups with preserved LVEF without CAD in the LOOP study.

	Hypertension	Diabetes	Obese
	n = 1,024	n = 307	n = 272
Global longitudinal strain			
• Normal	896 (90)	263 (90)	229 (88)
• Abnormal	96 (10)	31 (11)	30 (12)
Septal e’			
• Normal	336 (33)	101 (33)	93 (34)
• Abnormal	687 (67)	206 (67)	178 (66)
Lateral e’			
• Normal	172 (17)	45 (15)	35 (13)
• Abnormal	850 (83)	260 (85)	236 (87)
E/e’			
• Normal	936 (92)	268 (88)	241 (90)
• Abnormal	83 (8)	37 (12)	29 (10)
E/A			
• Normal	1,013 (99.5)	305 (100)	269 (99.6)
• Abnormal	5 (0.5)	0 (0)	1 (0.4)
Tricuspid regurgitation velocity			
• Normal	598 (89)	149 (87)	131 (85)
• Abnormal	76 (11)	22 (13)	23 (15)
Left atrial volume			
• Normal	928 (91)	280 (91)	249 (92)
• Abnormal	96 (9)	27 (9)	23 (9)
Left atrial reservoir strain			
• Normal	941 (93)	283 (94)	237 (89)
• Abnormal	70 (7)	19 (6)	29 (11)

LV: left ventricular; LVEF: left ventricular ejection fraction; CAD: coronary artery disease.

**Table 5:** Subclinical LV and LA abnormalities in subgroup with normal LAVi and LVEF and no CAD in the LOOP study.

	Whole population n = 1,030	Hypertension n = 928	Diabetes n = 280
Global longitudinal strain			
• Normal	900 (91)	813 (90)	239 (90)
• Abnormal	94 (10)	86 (10)	28 (11)
Left atrial reservoir strain			
• Normal	964 (95)	867 (95)	260 (95)
• Abnormal	52 (5)	49 (5)	15 (6)

LV: left ventricular; LAVi: left atrial volume index; LVEF: left ventricular ejection fraction; CAD: coronary artery disease.

As for LVEF, abnormal systolic dysfunction by GLS was more frequent in participants with previous acute myocardial infarction (44% vs. 15%, p<0.001) and known heart failure (71% vs. 16%, p<0.001).

### Left ventricular diastolic function

Among the 1,441 participants included in the study 393 (27%) had DDF (Table 2). Of these, 280 (71%) had DDF grade 1, 41 (10%) had DDF grade 2, and 10 (3%) had DDF grade 3. Additionally, 62 (16%) had indeterminate grade of DDF due to either missing or conflicting filling pressure indicators.

Impaired myocardial relaxation was a frequent finding in these participants as well as in the CCHS participants, whereas other markers of elevated filling pressure (high TR velocity, dilated LA, and elevated E/e’) were noted in approximately 10% of participants and slightly less frequently than in the CCHS (Table 2). Impaired relaxation and markers of elevated filling pressure were unchanged when restricting the analysis to participants without CAD (Table 3).

### Left atrial structure and function

By conventional LA assessment, 149 (10%) exhibited a dilated LA with the majority having mildly dilated LA (77%). LA speckle tracking was feasible in 1,421 (99%) of the population (feasibility in both projections: n = 1,347; one projection only: n = 74). Mean frame rate was 67±8 frames per second. Abnormal reservoir strain was observed in 130 (9%) of participants, and in 7% of those without CAD (Table 3), which was consistent among those with normal LVEF and no CAD. Notably, in this subgroup, the obese participants more frequently exhibited abnormal reservoir strain (Table 4). Abnormal reservoir strain was further observed in 7% of participants with normal LA size, and in 5% of those with normal LVEF, normal LA size and no CAD (Table 5). Of note, abnormal LA size and reservoir strain was more frequently observed in the CCHS (Table 2).

**Fig 3:**
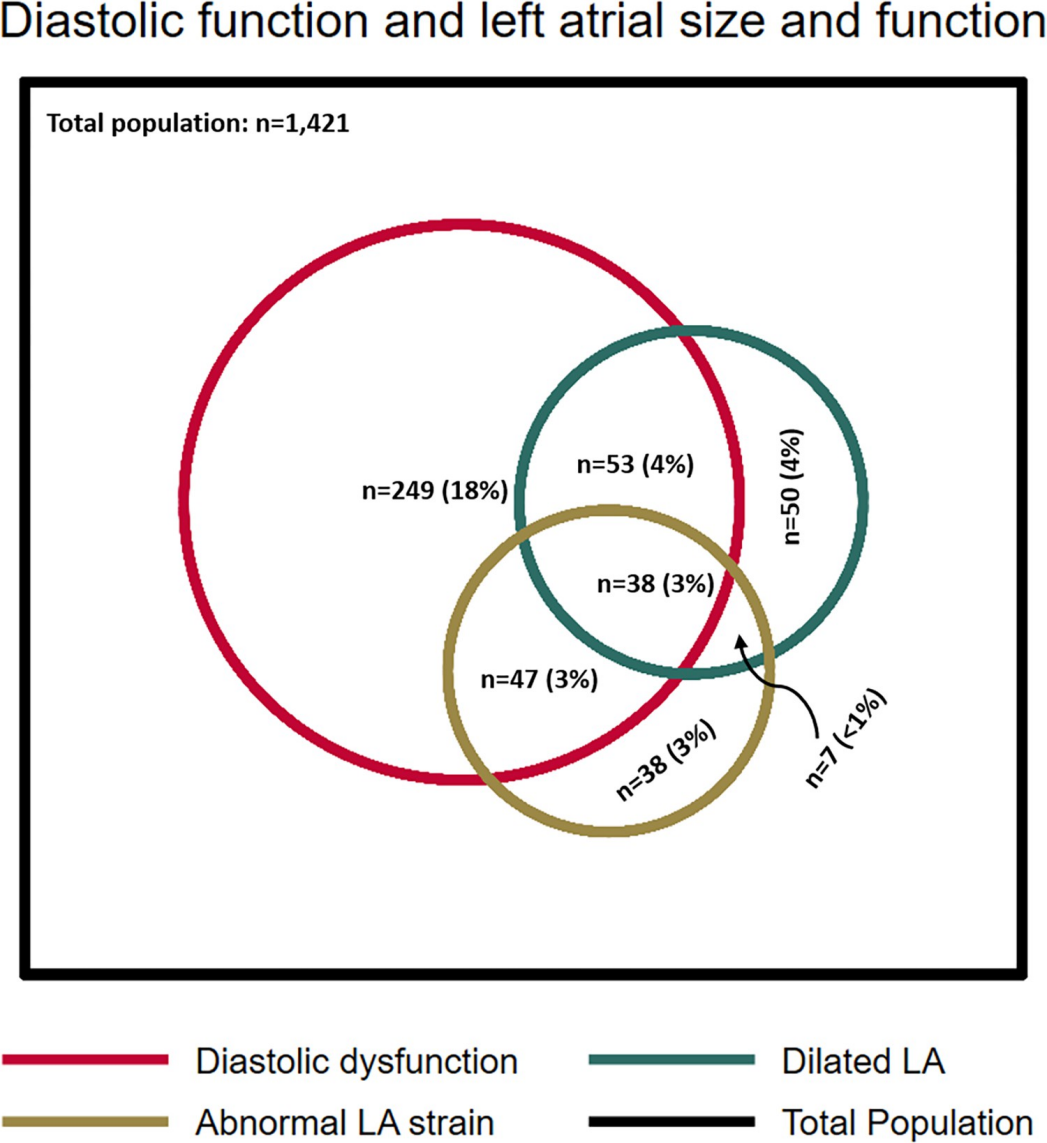
Diagram of diastolic dysfunction and abnormal left atrial size and function. The figure shows the prevalence of diastolic dysfunction, dilated LA, and abnormal LA strain as well as the overlap between the different groups. The percentages represent the proportion according to the total population in whom LA strain was obtainable (n = 1,421). LA: left atrium.

Both abnormal LA size and abnormal LA strain was observed in approximately a fifth of the participants with DDF (23% and 22%, respectively). The overlap between abnormalities in LA size, LA function and diastolic function is shown in Fig 3.

### Right ventricular function and pressure

Tricuspid regurgitant gradient was obtainable in 909 (63%) participants. Of these, 43 had chronic obstructive pulmonary disease. In participants without known pulmonary vascular disease (n = 866), 92 (11%) had either an intermediate or high likelihood of pulmonary hypertension. The likelihood of pulmonary hypertension increased with increasing risk group and so did the tricuspid regurgitant gradient (S3 and S4 Tables).

With respects to RV systolic function, 127 (9%) presented with reduced systolic function. Abnormal RV systolic function was more frequently observed in participants with prior acute myocardial infarction (23% vs. 7%, p<0.001), participants with known heart failure (26% vs. 8%, p<0.001), and participants with COPD (n = 88) (15% vs. 8%, p = 0.042).

### Valvular abnormalities

Valvular calcification was frequently observed, with 36% of participants having aortic valve sclerosis and 27% having mitral annular calcification. Significant valvular disease–moderate or severe MR, MS, AR, or AS–was observed in 71 (5%) of the participants. The majority of these comprised of aortic valve abnormalities, whereas significant mitral valve abnormalities were not frequently observed (Fig 1) (S3 Table). Participants with aortic valve calcification (sclerosis with or without stenosis) more frequently had DDF (31% vs. 25%, p = 0.033), abnormal GLS (22% vs. 15%, p<0.001), and more frequent abnormal LA strain (12% vs. 7%, p = 0.004).

Participants with mitral annular calcification more frequently exhibited DDF, abnormal E/e’, abnormal LA size, and abnormal LA strain compared to those without mitral annular calcification (Table 6).

**Table 6:** LV functional abnormalities according to MAC in the LOOP study.

	No MAC	MAC	P-value
	n = 1,049	n = 392	
Diastolic function			<0.001
• Normal	708 (68)	215 (55)	
• Diastolic dysfunction	259 (25)	134 (34)	
• Indeterminate	82 (8)	43 (11)	
Left ventricular ejection fraction			0.47
• Normal	925 (88)	351 (90)	
• Abnormal	124 (12)	41 (11)	
Global longitudinal strain			0.32
• Normal	831 (83)	301 (81)	
• Abnormal	173 (17)	73 (20)	
Septal e’			0.20
• Normal	318 (30)	105 (27)	
• Abnormal	729 (70)	285 (73)	
Lateral e’			0.07
• Normal	175 (17)	50 (13)	
• Abnormal	871 (83)	339 (87)	
E/e’			<0.001
• Normal	996 (95)	307 (79)	
• Abnormal	48 (5)	81 (21)	
E/A			0.89
• Normal	1,033 (99)	387 (99)	
• Abnormal	10 (1)	4 (1)	
Tricuspid regurgitant velocity			0.39
• Normal	596 (90)	215 (88)	
• Abnormal	68 (10)	30 (12)	
Left atrial volume			0.005
• Normal	955 (91)	337 (86)	
• Abnormal	94 (9)	55 (14)	
Left atrial reservoir strain			<0.001
• Normal	958 (93)	333 (86)	
• Abnormal	77 (7)	53 (14)	

LV: left ventricular; MAC: mitral annular calcification.

### Cardiac structure and function by accumulating risk factors

Cardiovascular risk factors and a detailed outline of the cardiac abnormalities observed in the entire population and stratified according to CHA_2_DS_2_-VASc score are listed in S3 Table and all individual echocardiographic measures are listed in S4 Table.

For the LV, neither LVMI nor RWT increased with higher risk category by the CHA_2_DS_2_-VASc score, however, a higher degree of LV remodeling was observed in those with CHA_2_DS_2_-VASc≥6. While GLS worsened with higher risk category, LVEF did not. However, both abnormal GLS and LVEF became more frequent with higher CHADS-VASc score.

Both early and late transmitral inflow velocities increased with increasing number of clinical risk factors, however, neither E-wave deceleration time nor E/A ratio differed between risk groups. Early myocardial relaxation velocity (e’) decreased significantly with increasing risk score and correspondingly E/e’ increased with increasing number of risk factors (S4 Table). DDF became exceedingly more prevalent with increasing clinical risk score, and so did the severity of DDF since DDF grade 1 became less prevalent and grade 2 concordantly more prevalent with increasing clinical risk score, whereas grade 3 was unchanged.

Abnormal LA size became slightly more frequent with increasing risk group (S3 Table). Abnormal LA strain became more frequent with higher risk score (S3 Table). All LA speckle tracking measures as well as volume-based functional LA measurements decreased significantly with increasing clinical risk score. Similarly, both LAVmax and LAVmin increased with increasing risk score, specifically in those with highest risk score (S4 Table).

Presence of RV systolic dysfunction increased with higher risk group (S3 Table), and TAPSE decreased accordingly (S4 Table).

Although significant valve disease did not become more frequent with increasing risk score, presence of mitral annular calcification and mild AR became more prevalent with higher CHA_2_DS_2_-VASc score.

## Discussion

The present report provides a detailed characterization of the echocardiographic phenotype of elderly participants from the general population with risk factors for AF and stroke, and specifically the cardiac structure and function. It provides an overview of the most frequently encountered cardiac abnormalities. It is notable that clinically relevant structural heart disease (significant valve disease and LVH) and reduced LVEF was only observed in a minor subset of the participants, even though the LOOP study found a third of participants to develop SCAF [5]. This emphasizes the need for applying novel techniques to detect subclinical myocardial dysfunction. In fact, our findings highlight the potential of such measures as speckle tracking could detect systolic dysfunction to a greater extent than LVEF. While abnormal LA strain was as frequently encountered as abnormal LA size, abnormal LA size and function did not frequently co-exist, suggesting that these two aspects could complement each other for the evaluation of the LA.

### Left ventricular structure

Structural abnormalities of the LV were frequently observed in our study with a prevalence of 40% but this was driven by concentric remodeling with a prevalence in-between what has been observed in other community-based cohort studies of elderly (33% in our study vs. 19% of elderly by Selmeryd et al. [22] vs. 36% in the ARIC study [23]). However, findings from Selmeryd et al. suggest that concentric remodeling may simply be an innocent bystander and does not confer an increased risk of outcome in elderly [22]. This is in contrast to abnormal LV mass and LVH which is associated with cardiovascular events in the elderly [22, 24]. LVH may therefore be more interesting in terms of prognosis. LVH was not frequent in our study (6%), and lower than in other population-based studies [25–27]. It is however worth noting that comparability to other studies is difficult as the presence of LVH is highly dependent on the definition. This was emphasized in The Helsinki Aging Study, which showed that LVH ranged between 36 to 67% of participants > 75 years of age depending on the LVH definition [25]. Interestingly, even the healthy subgroup in the Helsinki Aging Study exhibited a high prevalence of LVH (as high as 20% depending on criteria). The differences in our findings compared to other studies are unclear but may reflect the small cavity size observed in elderly which could affect the calculation of LV mass by Devereux’ formula. Additional 3-dimensional analysis, which has shown accuracy for LV mass estimation, will help clarify whether the low prevalence of LVH observed in this study was due to such technical issues rather than population sampling [28].

The CCHS is more comparable to our study in terms of ethnic background. We observed that LV mass was lower in the LOOP study compared to the CCHS, which may reflect that the CCHS participants were older and more frequently had hypertension.

### Left ventricular function

LV systolic dysfunction by LVEF was more frequent as compared to other population-based studies, which have generally observed a prevalence of 1–2% [29]. However, estimates from the Rotterdam Study revealed a higher prevalence of systolic dysfunction with increasing age, afflicting around 5–10% of elderly aged above 75 years of age [30], and this may even be an underestimate as they defined systolic dysfunction by fractional shortening which is less sensitive than LVEF for detecting systolic dysfunction. However, the ARIC study also reported abnormal LVEF in approximately 10%, which is comparable to our findings [31]. Interestingly, the prevalence of abnormal GLS in ARIC was around 12%, which is lower than our observation of 18%. However, this discrepancy may be largely explained by the differences in cut-off values (15% in ARIC and 16% in this study) and the software utilized. It is worth noting that abnormal GLS was not an infrequent finding when LVEF was preserved, whereas abnormal LVEF was seldomly observed when GLS was normal, supporting GLS’ application to detect subclinical LV systolic dysfunction. While GLS has shown to be a powerful predictor of outcome in patients with overt heart disease [32], it has also shown promise in at-risk patients [29, 33]. Shah and Solomon outlined the evolution of systolic function from a preclinical stage–in the presence of risk factors–to the development of overt heart disease [34]. They suggested that GLS represents an early marker of systolic dysfunction, whereas LVEF is largely preserved at a preclinical stage due to an augmentation of circumferential systolic function. Indeed, we observed that approximately 10% of participants with preserved LVEF and without CAD exhibited abnormal GLS, emphasizing its potential as an early marker of end-organ damage in at-risk conditions such as hypertension and diabetes.

DDF was fairly prevalent in this study and was present in approximately a fourth of the population. However, direct comparability to other studies is very challenging as the approach for assessing diastolic function differs substantially across population studies [26, 35–37]. Furthermore, the recommendations for DDF have changed within the recent years with a considerably different approach than earlier recommendations [19, 38].

### Left atrial abnormalities

LA size and function are of particular interest in the LOOP study as the purpose is prevention of stroke through subclinical AF detection. The LOOP study found 32% of high-risk elderly to have subclinical AF [5]. In that regard, it is worth noting that only 10% in our study had a structurally abnormal LA by current guideline definitions. This is slightly lower than the prevalence observed in the ARIC study [39], and suggests that other markers of LA dysfunction may be needed for predicting subclinical AF. To that end, it should be noted that magnetic resonance imaging findings from the LOOP study have found that measures of LA function in particular may improve clinical risk scores for prediction of subclinical AF [40], and atrial function as determined by echocardiography has likewise shown potential for predicting subclinical AF [9, 41]. In that regard, it is interesting that 7% of those with normal LA size did exhibit abnormal LA function by speckle tracking analyses. However, it should be emphasized that abnormal LA strain was not overall more frequently noted than abnormal LA size. Even so, it is interesting to note that abnormal LA strain and abnormal LA size did not necessarily co-exist, which stresses that LA strain and size should be used in concert to assess prognosis and DDF. Indeed, we observed that it was not possible to assess presence of DDF in 9% of the participants. LA strain may be valuable in this regard as previously outlined by Singh et al. [42]. Findings from the CCHS has previously supported the use of LA strain a prognostic marker of cardiovascular morbidity and mortality in the general population [43], irrespective of cardiovascular risk profile, even in individuals with normal LA size, further emphasizing its potential as a subclinical marker of LA dysfunction of potential value in the general population.

It is also noteworthy that abnormal LA strain was most frequently noted in obese participants, a patient group in which LA volume may be unreliable, and in which there is a need for another approach for evaluating the LA [44].

### Right ventricular function

The most frequently applied metric of RV systolic performance is the TAPSE, which has been associated with outcome in the general population [45]. Even though a TAPSE below 17 mm is considered abnormal, it is worth noting that a TAPSE below 24 mm is associated with adverse outcomes [11, 45]. The guidelines may therefore be underestimating the proportion of participants with abnormal RV function. This is also supported by a substudy from the ARIC study which looked into RV function by 3-dimensional echocardiography and observed nearly a fifth of elderly people to have RV dysfunction [46], which is approximately double of what we observed.

The prevalence of pulmonary hypertension is uncertain as the assessment relies on a visible tricuspid regurgitation for estimation of right ventricular pressure. Based on 2,823 participants in the Rotterdam Study, pulmonary hypertension was prevalent in 2.6% and appeared more frequently with higher age (8.3% when age is above 85 years) [47]. We estimated an intermediate-high likelihood of pulmonary hypertension in 6.8% which is comparable to the Rotterdam study.

### Valvular disease

Valvular heart disease becomes exceedingly more frequent with age. By echocardiographic screening, the Tromsø study found a prevalence of significant valve disease of 3.3% [48]. This falls well in line with our observation. In contrast, however, the OxValve population study found that valve disease was present in more than half of participants aged > 65 years of age, with significant left-sided valve disease observed in 6.4% [49]. Our finding of a high proportion of participants with aortic sclerosis is consistent with the OxValve study. This is an important finding since aortic sclerosis is associated with an increased risk of cardiovascular outcomes in the elderly [50], and might be amenable to risk factor modification. Risk factor modification commenced in middle-age appears beneficial in terms of slowing down the progression of aortic sclerosis [51]. Whether risk factor modification is also beneficial when initiated in older age is unknown. Furthermore, aortic sclerosis may progress to aortic valve stenosis that could warrant surgical intervention. Given the improvements in transcatheter intervention, elderly people are now more frequently considered eligible candidates for aortic valve replacement. However, it should be noted that significant AS was not frequently observed in this study (0.90%). In fact, it was less frequently observed compared to the National Echocardiographic Database of Australia (NEDA) which reported severe AS in 6.3% [52]. The discrepancy may rely on the fact that NEDA represents a clinical registry, meaning they reported findings from echocardiograms performed for a clinical indication. Accordingly, the prevalence of significant AS in our study falls well in line with that reported in the ARIC study of 0.7% [51]. Even though some have argued for opportunistic screening for aortic valve disease [53], the cost-benefit of screening and following elderly at-risk patients with aortic sclerosis in unclear. Follow-up echocardiographic examinations in the LOOP study have now been concluded and may help to illustrate the extent to which aortic sclerosis progresses to AS in these participants.

Mitral annular calcification was a frequent finding in the participants. Previous population-based studies have reported a prevalence between 5 and 42% [54–57], depending on the imaging modality used and the age of the population. Since mitral annular calcification has been linked to an increased risk of stroke [57], it is striking that abnormal LA size and mechanics was more frequently noted in those with mitral annular calcification, indicating more frequent atrial dysfunction in this condition, which may be a contributing factor for the development of stroke.

### Strengths and limitations

The primary strengths of this study are the large sample size, prospective design as part of a randomized controlled trial, and the rigorous echocardiographic protocol for acquisition and analyses. However, some limitations do apply. Since participants were recruited from the general population by letter invitation, the study may be subjected to healthy user bias as the healthier participants would be more prone to participate in the study. Echocardiography was only performed in a subset of the participants due to logistic reasons, however, these participants were comparable to those who did not undergo echocardiography, which speaks against significant selection bias. Initially, the inclusion focused on those with an ILR, and later also included participants from the control group resulting in oversampling of participants in the ILR group, and hence, a distribution of participants discordant from the main LOOP study. However, as shown in the supplemental material this did not influence the extent of cardiac abnormalities observed in the sampling cohort for this substudy as the findings were balanced between the randomization group.

Finally, we may be overestimating the prevalence of DDF as we applied the cut-offs and classification scheme recommended in guidelines even though elderly exhibit benign age-related declines in diastolic measures such as the e’. Clinically it may be more sensible to employ a more age-based approach for assessing diastolic function [35].

## Conclusion

Cardiac abnormalities are frequently recognized in elderly participants at high-risk of stroke. Valvular calcification, left ventricular geometrical changes, and aortic regurgitation are the most frequently encountered irregularities, observed in around 20–40% of participants. Left ventricular systolic dysfunction is observed in one-tenth of participants by conventional measures, whereas advanced echocardiographic techniques detect systolic dysfunction nearly twice as often. Finally, LA remodeling is observed in 10%. This is in contrast to the LOOP study finding 32% of participants to develop subclinical AF, which emphasizes the need for more comprehensive evaluation of the LA, including assessment of LA function. Future echocardiographic substudies of the LOOP study will relate these findings to incident subclinical AF, AF burden, and cardiovascular outcomes.

## Supporting information

S1 Table Clinical characteristics according to substudy inclusion. (DOCX)

S2 Table Echocardiographic abnormalities according to randomization group. (DOCX)

S3 Table Cardiac risk factors and abnormalities according to clinical risk score. (DOCX)

S4 Table Echocardiographic characteristics according to clinical risk score. (DOCX)
