# Supplementary material for: Potential role of conventional and speckle-tracking echocardiography in the screening of structural and functional cardiac abnormalities in elderly individuals: Baseline echocardiographic findings from the LOOP study
Source: PLoS One. 2022 Jun 3;17(6):e0269475. doi: 10.1371/journal.pone.0269475 (PMC9165786; doi:10.1371/journal.pone.0269475)
Supplement: S1 Table — (DOCX) [file pone.0269475.s001.docx]

**Supplementary table 1 – Clinical characteristics according to substudy inclusion**

|  | **No echocardiogram n=4,565** | **Echocardiogram n=1,441** | **p-value** |
| --- | --- | --- | --- |
| Age, years | 74.8±4.1 | 74.4±4.1 | <0.001 |
| Men, n (%) | 2,383 (52.2) | 784 (54.4) | 0.15 |
| Smoking pack years | 6 [0;28] | 7 [0;28] | 0.70 |
| Alcohol consumption, units/week | 5 [1;10] | 5 [1;11] | 0.13 |
| Systolic blood pressure, mmHg | 150.2±19.6 | 149.3±18.9 | 0.15 |
| Diastolic blood pressure, mmHg | 83.9±11.3 | 84.8±11.1 | 0.014 |
| Height, cm | 170.3±8.8 | 171.0±8.9 | 0.011 |
| Weight, kg | 80.5±15.6 | 81.0±15.6 | 0.36 |
| Body mass index, kg/m^2^ | 27.7±4.6 | 27.6±4.5 | 0.56 |
| Heart rate, beats/minute | 71.3±12.5 | 71.7±12.3 | 0.36 |
| Hypertension, n (%) | 4,138 (90.7) | 1,306 (90.6) | 0.95 |
| Diabetes mellitus, n (%) | 1,289 (28.2) | 421 (29.2) | 0.48 |
| Heart failure, n (%) | 201 (4.4) | 65 (4.5) | 0.86 |
| Previous stroke or systemic embolism n (%) | 1,008 (22.1) | 302 (21.0) | 0.36 |
| Previous myocardial infarction, n (%) | 473 (10.4) | 148 (10.3) | 0.92 |
| Previous CABG, n (%) | 251 (5.5) | 86 (6.0) | 0.50 |
| Known valve disease, n (%) | 185 (4.1) | 59 (4.1) | 0.95 |
| PAD, n (%) | 301 (6.6) | 91 (6.3) | 0.71 |
| COPD, n (%) | 352 (7.7) | 88 (6.1) | 0.041 |

Gaussian-distributed continuous variables are presented as mean±SD. Non-gaussian-distributed variables are presented as median with interquartile range.
Abbreviations: CABG: coronary artery bypass grafting; PAD: peripheral artery disease; COPD: chronic obstructive pulmonary disease
