# Supplementary material for: Potential role of conventional and speckle-tracking echocardiography in the screening of structural and functional cardiac abnormalities in elderly individuals: Baseline echocardiographic findings from the LOOP study
Source: PLoS One. 2022 Jun 3;17(6):e0269475. doi: 10.1371/journal.pone.0269475 (PMC9165786; doi:10.1371/journal.pone.0269475)
Supplement: S2 Table — (DOCX) [file pone.0269475.s002.docx]

**Supplementary table 2 – Echocardiographic abnormalities according to randomization group**

|  | Control group  (n=421) | ILR group  (n=1,020) | p-value |
| --- | --- | --- | --- |
| **Left ventricle** |  |  |  |
| Left ventricular structure, n (%) - Normal geometry - Concentric remodeling - Eccentric hypertrophy - Concentric hypertrophy | 252 (60) 140 (33) 16 (4) 13 (3) | 616 (61) 340 (33) 22 (2) 41 (4) | 0.29 |
| Systolic function by LVEF, n (%) - Normal - Mildly reduced - Moderately reduced - Severely reduced | 379 (90) 36 (9) 4 (1) 2 (0.5) | 897 (88) 106 (10) 16 (2) 1 (0.1) | 0.26 |
| Systolic function by GLS, n (%) - Normal - Impaired | 331 (82) 75 (19) | 801 (82) 171 (18) | 0.70 |
| Diastolic function, n (%) - Normal - Indeterminate - Diastolic dysfunction | 267 (63) 37 (8) 117 (28) | 656 (64) 88 (9) 276 (25) | 0.95 |
| **Left atrium** |  |  |  |
| Left atrial size, n (%) - Normal size - Mildly dilated - Moderately dilated - Severely dilated  Left atrial reservoir strain, n (%) - Normal - Abnormal | 372 (88) 37 (9) 7 (2) 5 (1)  381 (91) 36 (9) | 920 (90) 77 (8) 11 (1) 12 (1)  910 (91) 94 (9) | 0.68  0.66 |
| **Right ventricle** |  |  |  |
| Systolic function, n (%) - Normal - Impaired | 389 (93) 31 (7) | 923 (91) 96 (9) | 0.21 |
| Right ventricular pressure, n (%)* - Low likelihood of pulmonary hypertension - Intermediate likelihood of pulmonary hypertension - High likelihood of pulmonary hypertension | 248 (91) 25 (9) 1 (0.4) | 526 (89) 64 (11) 2 (0.3) | 0.75 |
| **Valvular function** |  |  |  |
| Mitral annular calcification, n (%)  Mitral regurgitation, n (%) - No or trivial MR - Mild MR - Moderate MR | 117 (28)   399 (95) 20 (5) 2 (0.5) | 275 (27)   969 (95) 50 (5) 1 (0.1) | 0.75  0.39 |
| Mitral stenosis, n (%) - No MS - Moderate MS | 421 (100) 0 (0) | 1,019 (100) 1 (0.1) | 1.00 |
| Aortic regurgitation - No or trivial AR - Mild AR - Moderate AR - Severe AR | 318 (76) 80 (19) 22 (5) 1 (0.2) | 836 (82) 151 (15) 32 (3) 1 (0.1) | 0.020 |
| Aortic stenosis - No AS or aortic sclerosis - Aortic sclerosis - Mild AS - Moderate AS - Severe AS | 261 (62) 141 (33) 15 (4) 4 (1) 0 (0) | 616 (60) 380 (37) 15 (1) 6 (0.6) 3 (0.3) | 0.06 |

ILR: implantable loop recorder; LVEF: left ventricular ejection fraction; GLS: global longitudinal strain; MR: mitral regurgitation; MS: mitral stenosis; AR: aortic regurgitation; AS: aortic stenosis.

*Likelihood based on individuals without known chronic obstructive pulmonary disease. Low likelihood: TR_Vmax_ <2.8m/s, intermediate likelihood: TR_Vmax_: 2.8-3.4m/s, high likelihood: TR_Vmax_>3.4m/s.
