# Supplementary material for: Potential role of conventional and speckle-tracking echocardiography in the screening of structural and functional cardiac abnormalities in elderly individuals: Baseline echocardiographic findings from the LOOP study
Source: PLoS One. 2022 Jun 3;17(6):e0269475. doi: 10.1371/journal.pone.0269475 (PMC9165786; doi:10.1371/journal.pone.0269475)
Supplement: S3 Table — (DOCX) [file pone.0269475.s003.docx]

**Supplementary table 3 – Cardiac risk factors and abnormalities according to clinical risk score**

|  | All  (n=1,441) | CHA_2_DS_2_-VASc: 2-3 n=708 | CHA_2_DS_2_-VASc: 4-5 n=623 | CHA_2_DS_2_-VASc≥6 n=110 | p-value |
| --- | --- | --- | --- | --- | --- |
| **Clinical characteristics** |  |  |  |  |  |
| Age, years | 74.4±4.1 | 73.0±3.1 | 75.4±4.4 | 77.2±4.7 | <0.001 |
| Men, n (%) | 784 (54) | 470 (66) | 269 (43) | 45 (41) | <0.001 |
| Hypertension, n (%) | 1,305 (91) | 632 (89) | 566 (91) | 107 (97) | 0.027 |
| Diabetes mellitus, n (%) | 421 (29) | 152 (22) | 218 (35) | 51 (46) | <0.001 |
| Heart failure, n (%) | 65 (5) | 14 (2) | 35 (6) | 16 (15) | <0.001 |
| Previous stroke or systemic embolism, n (%) | 302 (21) | 13 (2) | 197 (32) | 92 (84) | <0.001 |
| **Left ventricle** |  |  |  |  |  |
| Left ventricular geometry, n (%) - Normal geometry - Concentric remodeling - Eccentric hypertrophy - Concentric hypertrophy | 868 (60) 480 (33) 38 (3) 54 (4) | 441 (62) 230 (33) 16 (2) 20 (3) | 384 (62) 197 (32) 17 (3) 25 (4) | 43 (39) 53 (48) 5 (5) 9 (8) | <0.001 |
| Systolic function by LVEF, n (%) - Normal - Mildly reduced - Moderately reduced - Severely reduced | 1,276 (89) 142 (10) 20 (1) 3 (0.2) | 643 (91) 56 (8) 9 (1) 0 (0) | 541 (87) 68 (11) 11 (2) 3 (0.5) | 92 (84) 18 (16) 0 (0) 0 (0) | 0.020 |
| Systolic function by GLS, n (%) - Normal - Impaired | 817 (59) 246 (18) | 579 (85) 100 (15) | 475 (56) 118 (20) | 78 (74) 28 (26) | 0.003 |
| Diastolic function, n (%) - Normal - Indeterminate - Diastolic dysfunction | 923 (64) 125 (9) 393 (27) | 489 (69) 50 (7) 169 (24) | 382 (61) 64 (10) 177 (28) | 52 (47) 11 (10) 47 (43) | <0.001 |
| **Left atrium** |  |  |  |  |  |
| Left atrial size, n (%) - Normal size - Mildly dilated - Moderately dilated - Severely dilated  Left atrial reservoir strain, n (%) - Normal - Abnormal | 1,292 (90) 114 (8) 18 (1) 17 (1)  1,154 (81) 130 (9) | 627 (89) 65 (9) 13 (2) 3 (0.4)  64 (93) 51 (7) | 569 (91) 40 (6) 2 (0.3) 12 (2)  553 (90) 60 (10) | 96 (87) 9 (8) 3 (3) 2 (2)  90 (83) 19 (17) | 0.002  0.002 |
| **Right ventricle** |  |  |  |  |  |
| Systolic function, n (%) - Normal - Impaired | 1,312 (91) 127 (9) | 658 (93) 49 (7) | 559 (90) 64 (10) | 95 (87) 13 (13) | 0.031 |
| Right ventricular pressure, n (%)* - Low likelihood of pulmonary hypertension - Intermediate likelihood of pulmonary hypertension - High likelihood of pulmonary hypertension | 774 (89) 89 (10) 3 (0.4) | 370 (91) 38 (9) 0 (0) | 351 (90) 34 (9) 3 (0.8) | 53 (76) 17 (24) 0 (0) | 0.001 |
| **Heart valves** |  |  |  |  |  |
| Mitral annular calcification, n (%)  Mitral regurgitation, n (%) - No or trivial MR - Mild MR - Moderate MR | 392 (27)  1,368 (95) 70 (5) 3 (0.2) | 153 (22)  681 (96) 27 (4) 0 (0) | 190 (31)  584 (94) 36 (6) 3 (0.5) | 49 (45)  103 (94) 7 (6) 0 (0) | <0.001  0.10 |
| Mitral stenosis, n (%) - No MS - Moderate MS | 1,440 (99.9) 1 (0.07) | 708 (100) 0 (0) | 622 (99.8) 1 (0.2) | 110 (100) 0 (0) | 0.51 |
| Aortic regurgitation - No or trivial AR - Mild AR - Moderate AR - Severe AR | 1,154 (80) 231 (16) 54 (4) 2 (0.1) | 591 (84) 98 (14) 19 (3) 0 (0) | 476 (76) 113 (18) 33 (5) 1 (0.2) | 87 (79) 20 (18) 2 (2) 1 (1) | 0.004 |
| Aortic stenosis - No AS or aortic sclerosis - Aortic sclerosis - Mild AS - Moderate AS - Severe AS | 877 (61) 521 (36) 30 (2) 10 (0.7) 3 (0.2) | 449 (63) 240 (34) 14 (2) 4 (0.6) 1 (0.1) | 368 (59) 235 (38) 13 (2) 5 (0.8) 2 (0.3) | 60 (55) 46 (42) 3 (3) 1 (1) 0 (0) | 0.57 |

LVEF: left ventricular ejection fraction; GLS: global longitudinal strain; MR: mitral regurgitation; MS: mitral stenosis; AR: aortic regurgitation; AS: aortic stenosis
*Likelihood based on participants without known chronic obstructive pulmonary disease. Low likelihood: TR_Vmax_ <2.8m/s, intermediate likelihood: TR_Vmax_: 2.8-3.4m/s, high likelihood: TR_Vmax_>3.4m/s
