# Supplementary material for: Potential role of conventional and speckle-tracking echocardiography in the screening of structural and functional cardiac abnormalities in elderly individuals: Baseline echocardiographic findings from the LOOP study
Source: PLoS One. 2022 Jun 3;17(6):e0269475. doi: 10.1371/journal.pone.0269475 (PMC9165786; doi:10.1371/journal.pone.0269475)
Supplement: S4 Table — (DOCX) [file pone.0269475.s004.docx]

**Supplementary table 4 - Echocardiographic characteristics according to clinical risk score**

|  | All n=1,441 | CHA_2_DS_2_-VASc: 2-3 n=708 | CHA_2_DS_2_-VASc: 4-5 n=623 | CHA_2_DS_2_-VASc≥6 n=110 | p-value |
| --- | --- | --- | --- | --- | --- |
| **Left ventricular structure** |  |  |  |  |  |
| IVSd, cm | 0.93±0.17 | 0.92±0.17 | 0.92±0.17 | 0.97±9.16 | 0.017 |
| LVIDd, cm | 4.64±0.59 | 4.69±0.58 | 4.60±0.60 | 4.51±0.58 | 0.001 |
| LVPWd, cm | 0.90±0.17 | 0.90±0.16 | 0.90±0.17 | 0.95±0.18 | 0.016 |
| LVMI, g/m^2^ | 75±19 | 75±18 | 74±19 | 78±20 | 0.11 |
| RWT | 0.40±0.10 | 0.39±0.09 | 0.40±0.10 | 0.43±0.10 | 0.16 |
| LVEDV, mL | 86 (72;103) | 90 (75;107) | 82 (69;101) | 80 (68;94) | <0.001 |
| LVESV, mL | 33 (26;42) | 34 (27;43) | 31 (25;40) | 31 (24;39) | <0.001 |
| **Left ventricular systolic function** |  |  |  |  |  |
| LVEF, % | 61±7 | 61±7 | 60±7 | 60±7 | 0.16 |
| GLS, % | -18.3±2.7 | -18.5±2.6 | -18.2±2.8 | -17.8±3.0 | 0.016 |
| Systolic strain rate, %/s | -0.92±0.16 | -0.93±0.15 | -0.91±0.16 | -0.88±0.18 | 0.002 |
| Global s’, cm/s | 5.5±1.0 | 5.7±1.0 | 5.4±1.0 | 5.1±1.0 | <0.001 |
| **Left ventricular diastolic function** |  |  |  |  |  |
| E-wave, cm/s | 67±18 | 66±17 | 68±19 | 71±18 | 0.011 |
| A-wave, cm/s | 79±19 | 77±18 | 80±20 | 83±20 | <0.001 |
| E/A | 0.83 (0.69;1.00) | 0.84 (0.71;1.01) | 0.81 (0.68;1.00) | 0.81 (0.71;1.01) | 0.28 |
| E-wave deceleration time, ms | 227±62 | 225±61 | 228±62 | 231±67 | 0.51 |
| e’, cm/s | 7.1±1.7 | 7.3±1.7 | 6.9±1.7 | 6.6±1.5 | <0.001 |
| E/e’ | 9.8±3.2 | 9.3±2.6 | 10.2±3.5 | 11.2±4.2 | <0.001 |
| Early diastolic strain rate, %/s | 0.92±0.27 | 0.94±0.27 | 0.90±0.27 | 0.87±0.27 | 0.008 |
| Global e’, cm/s | 5.4±1.4 | 5.6±1.4 | 5.2±1.4 | 5.1±1.1 | <0.001 |
| **Right ventricle** |  |  |  |  |  |
| TAPSE, cm | 2.29±0.44 | 2.35±0.45 | 2.25±0.43 | 2.11±0.35 | <0.001 |
| TR_Vmax_, mmHg | 2.44±0.29 | 2.41±0.28 | 2.46±0.29 | 2.51±0.31 | 0.006 |
| **Left atrium** |  |  |  |  |  |
| LAVmax, mL/m^2^ | 24 (20;29) | 24 (19;29) | 23 (19;28) | 26 (20;30) | 0.040 |
| LAVmin, mL/m^2^ | 11 (8;14) | 11 (8;14) | 11 (8;15) | 13 (9;16) | 0.003 |
| Total LAEF, % | 52 (46;58) | 53 (47;59) | 52 (45;58) | 49 (43;56) | <0.001 |
| Passive LAEF, % | 25±9 | 25±8 | 24±9 | 23±8 | 0.015 |
| Active LAEF, % | 36±10 | 37±10 | 35±10 | 34±11 | <0.001 |
| LA reservoir strain, % | 34±8 | 35±9 | 33±8 | 31±8 | <0.001 |
| LA conduit strain, % | 14±5 | 15±6 | 14±5 | 13±5 | <0.001 |
| LA contraction strain, % | 19±6 | 20±7 | 19±6 | 18±6 | 0.002 |
| Late diastolic strain rate, %/s | 1.02±0.23 | 1.01±0.22 | 1.02±0.23 | 1.00±0.24 | 0.48 |
| Global a’, cm/s | 7.5±1.6 | 7.7±1.6 | 7.3±1.5 | 7.0±1.6 | <0.001 |

Continuous variables with normal distribution are shown as mean±SD, whereas continuous variables not showing normal distribution are shown as median with 25-75% percentiles.
IVSd: interventricular septal diameter; LVIDd: left ventricular internal diameter; LVPWd: left ventricular posterior wall diameter; LVMI: left ventricular mass index; RWT: relative wall thickness; LVEDV: left ventricular end-diastolic volume; LVESV: left ventricular end-systolic volume; LVEF: left ventricular ejection fraction; GLS: global longitudinal strain; TAPSE: tricuspid annular systolic plane excursion; TR_Vmax_: peak tricuspid regurgitant velocity; LAVmax: maximal left atrial volume; LAVmin: minimal left atrial volume; LAEF: left atrium emptying fraction; LA: left atrium
